# Simultaneous Determination of Isoniazid, Pyrazinamide and Rifampin in Human Plasma by High-performance Liquid Chromatography and UV Detection

**DOI:** 10.22037/ijpr.2019.1100849

**Published:** 2019

**Authors:** Behnam Dasht Bozorg, Ali Goodarzi, Fanak Fahimi, Payam Tabarsi, Nahid Shahsavari, Farzad Kobarfard, Farzaneh Dastan

**Affiliations:** a *Department of Clinical Pharmacy, School of Pharmacy, Shahid Beheshti University of Medical Sciences, Tehran, Iran. *; b *Chronic Respiratory Disease Research Center, National Research Institute of Tuberculosis and Lung Diseases (NRITLD), Shahid Beheshti University of Medical Sciences, Tehran, Iran.*; c *Phytochemistry Research Center, Department of Medicinal Chemistry, School of Pharmacy, Shahid Beheshti University of Medical Sciences, Tehran, Iran.*

**Keywords:** Anti-TB drugs, Therapeutic drug monitoring, HPLC-UV, Isoniazid, Pyrazinamide, Rifampin

## Abstract

Therapeutic Drug Monitoring (TDM) of first-line anti-tuberculosis (TB) drugs is a decisive tool, allowing the clinician to successfully treat TB patients. The objective of the study was to develop and optimize a simple, sensitive, and reliable high-performance liquid chromatography (HPLC) method for the simultaneous determination of isoniazid (INH), pyrazinamide (PZA), and rifampin (RIF) levels in human plasma. Nicotinamide was used as the internal standard and the samples were prepared after protein precipitation using acetonitrile and zinc sulfate. The separation was achieved using a C18 reversed-phase applying gradient elution. The mobile phase was a combination of water–methanol solution with a ratio of 95:05 (v/v) at the initial phase. All calibration curves had good linearity (r^2 ^> 0.99) and the inter- and intra-day RSDs were lower than 15%. The limit of detection with a signal-to-noise ratio (S/N) of 3:1 was 0.16, 0.5, and 0.33 μg mL^–1^ for INH, PZA, and RIF, respectively. The method presented here was selective, sensitive, and reproducible, and could be used for‌ therapeutic drug monitoring in the patients who were under treatment with these drugs.

## Introduction

Tuberculosis (TB) is the oldest documented infectious disease which is caused by the bacillus *Mycobacterium tuberculosis (M. tuberculosis). *Despite the availability of highly efficacious treatment for decades, TB remains a major global health problem. In 1993, the World Health Organization (WHO) declared TB, a global public health emergency. In 2010, there were estimated 8.5–9.2 million disease cases and 1.2–1.5 million deaths. TB is the second leading cause of death among infectious diseases worldwide (1, 2). Chemotherapy is designed to kill tubercle bacilli rapidly, to minimize the potential for the organisms to develop drug resistance, and to sterilize the host’s tissues. The achievement of these goals requires that a combination of agents with specific activities be administered for a sufficiently long period of time. Currently, there are 10 drugs approved by the United States Food and Drug Administration (FDA) for treating tuberculosis. Of the approved drugs isoniazid (INH), rifampin (RIF), ethambutol (EMB), and pyrazinamide (PZA) are considered as first-line antituberculosis agents and form the core of initial treatment regimens (3, 4). Therapeutic drug monitoring (TDM) is a standard clinical technique used for many disease states, including many infectious diseases. The use of TDM in the setting of tuberculosis (TB) allows the clinician to make informed decisions regarding the timely adjustment of drug therapy. Such adjustments may not be required for otherwise healthy individuals who are responding to the standard, four-drug TB regimens. However, the patients may be slow respondents to treatment due to infected with drug-resistant TB, in risk of drug-drug interactions or with concurrent disease states that significantly complicate the clinical situation. Such patients may benefit from TDM and early interventions may preclude the development of further drug resistance (5, 6). TDM can also be helpful in ensuring that the drug delivery is adequate (7, 8). Low anti-TB drugs’ concentrations are common and associated with relapse, treatment failure, and resistance in HIV-infected patients, resulting in poor clinical response to chemotherapy (9). Therefore, the measurement of anti-TB drugs’ plasma concentrations seems to be essential (8-10). 

In TDM studies, the assays should be very specific for the drugs of interest. In general, high-performance liquid chromatography (HPLC) and gas chromatography (GC) are preferred. Commonly used detection systems (ultraviolet or fluorescence detection for HPLC, mass spectrometry for HPLC or GC) work well, provided that the extensive interference checks are performed prior to validating the assays (5). Unsalan *et al.* have reported the determination of isoniazid, pyrazinamide, and rifampin in human plasma using an HPLC-UV method on a C-18 reverse phase stationary phase. However, their method requires two independent sample preparations and injections: one for PZA and RIF analysis and one for INH. Another HPLC method has been reported for simultaneous determination of PZA, RIF, and INH by Zhou *et al.* in which, separation has been performed on a Max-RP C12 column using an ion pair mechanism. A validated LC-Mass method has also been reported by Song *et al.* for simultaneous determination of PZA, INH, and RIF (11-13). To the best of our knowledge there has been no report for simultaneous determination of PZA, INH, and RIF in a single chromatographic run using C18 RP stationary phase coupled to a UV detector. In this study we developed and validated a simple and rapid method for the simultaneous measurement of serum concentrations of INH, RIF, and PZA. The method presented here has many advantages: it has a high level of sensitivity, accuracy and precision; a very fast and simple sample preparation is involved; and also this is for the first time which the simultaneous determination of the three drugs has been performed using a C18 Column coupled to a UV detector.

## Experimental

*Chemicals and Solvents*

Isoniazid, rifampin, and pyrazinecarboxamide were purchased from Merck Chemical Company (Darmstadt, Germany). Nicotinamaide (NA) was used as Internal Standard (IS) and was purchased from Sigma-Aldrich (Germany). Zinc sulfate 7H_2_0 was used for plasma’s protein precipitation and was also provided by Merck (Darmstadt, Germany). HPLC gradient grade methanol and acetonitrile were also obtained from Merck Chemical Co. (Darmstadt, Germany). Deionized water was obtained from Overseas Equipment and Services (OES) Inc. Water Purification System (Oklahoma, USA).

*Equipment and Chromatographic Conditions*

The HPLC system used was an Agilent Technologies 1200 series system (Germany) equipped with a binary gradient pump, an online degasser, an ultraviolet DAD detector, and an auto sampler. Chromatographic separation was carried out using the following column and mobile phases: revers phase C18 (250 mm × 4.6 mm, perfectSil Target ODS-3 5 μm particle size, MZ Analytical, Germany). The mobile phase was a gradient of water (solvent A) and methanol (solvent B). 

The composition of the initial mobile phase was (A:B) 95:05 v/v at a constant flow rate of 1.5 mL min^-1^ for 12 min, followed by a linear gradient to 20:80 v/v until 15 min and was kept until 23 min. At 23 min the mobile phase was changed to the initial composition and was maintained until 26 min before the next injection. The injection volume was 100 μL. The monitoring wavelengths were 254 nm for INH, PZA, NA, and 336 nm for RIF.

*Preparation of Standard Solutions and Quality Control Samples*

Stock solutions of INH, PZA, RIF, and NA at a concentration of 1.00 mg mL^-1^ were prepared in water, except for RIF which methanol was used instead. In order to obtain the desired concentrations, different volumes of the stock solutions were combined and diluted with water and methanol in case of rifampin. All of the solutions were stored at -20 °C.

Calibration standards of 0.5, 1.0, 2.5, 5.0, 10.0, 20.0 μg mL^-1^ of INH; 1.5, 3.0, 7.5, 15.0, 30.0, 60.0 μg mL^-1^ of PZA and 1.0, 2.0, 5.0, 10.0, 20.0, 40.0 of RIF were constructed by adding 45 μL of the standard solutions (5 μL of INH, 30 μL of PZA, and 10 μL of RIF of each prepared concentration) and 5 μL of the internal standard solution to 450 μL of a blank plasma.

Quality control (QC) samples were prepared using blank plasma at three different sets of concentrations: low (2.0 μg mL^-1^ of INH, 6.0 μg mL^-1^ of PZA and 4.0 μg mL^-1^ of RIF), middle (5.0 μg mL^-1^ of INH, 15.0 μg mL^-1^ of PZA and 10.0 μg mL^-1^ of RIF) and high (12.0 μg mL^-1^ of INH, 36.0 μg mL^-1^ of PZA and 24.0 μg mL^-1^ of RIF).

*Sample Preparation*

To an aliquot of 495 μL of plasma, NA solution (5 μL) as internal standard was added. The plasma samples were either fresh or thawed at the room temperature. They were then mixed with 40 μL of acetonitrile, 160 μL of zinc sulfate (10% in H_2_O) and 5 μL of ammonia (25%) separately. 

The samples were vigorously mixed using a vortex shaker for 1 min after each addition, and then were centrifuged at 14000 rpm for 10 min while the temperature was kept at 5 °C. 

The clear supernatants were stored at 5 °C due to the instability of rifampin at room temperature (14). Five minutes prior to injection, each one of the samples was placed out of the cooling system to reach the room temperature and then was injected (100 μL) into the HPLC system. The sample preparation should be as quick as possible due to the rifampin instability at the ambient temperature.

*Stability*

The short-term stability of the target compounds in the plasma was evaluated by leaving QC samples at 4 °C temperature for 24 h. The long-term stability was determined by storing QC samples at 4 °C for 1 week. All QC samples for stability testing included low, middle, and high concentrations, and each concentration had three repeats. During each analytical run, a standard curve was constructed to calculate the concentration of the target compounds. QC samples were also used to determine the stability of the analytes after three times of freeze and thaw processes.

## Results

*Method Validation*

To confirm the applicability of this method, the linearity of the calibration curve, precision, accuracy, and limits of quantification and detection (LOQ and LOD) were determined for the analysis of INH, PZA, and RIF.

All calibration standards were prepared using the procedure mentioned above and the integrated peak areas were used to draw calibration curves.

*Linearity*

Calibration graphs were constructed by six spiked plasma samples ranging from 0.5 to 20.0 μg mL^-1^ for INH, 1.5 to 60.0 μg mL^-1^for PZA, and 1.0 to 40.0 μg mL^-1^ for RIF. Each calibration curve was constructed by plotting the peak area ratio of the drug to the internal standard versus the nominal concentrations of the samples. Linearity was assured by calculating the regression coefficients for the calibration curves. Range of the analysis was determined by assuring that all the points on the calibration curve were characterized by acceptable precision and accuracy.

*Precision and Accuracy*

Precision and accuracy were determined by the analysis of QC samples spiked at three concentration levels listed above. The intra-day precision and accuracy were determined by replicate analysis of a QC sample at each concentration (n = 3). To determine the inter-day precision and accuracy, three consecutive batches of QC samples were made by the same procedure on three different days. The calculated mean concentrations relative to the nominal concentrations (relative error: RE%) were used to express accuracy and relative standard deviations (RSD%) of the retention time and the area of the target compounds and IS were used to estimate the inter- and intra-day precision. Figures of merit for the validated method are presented in Table 1.

*LOD and LLOQ*

The lower limit of quantification (LLOQ) was defined as the lowest concentration on the calibration curve that can be determined with an accuracy of 80-120% and a precision of less than 20%. The limit of detection (LOD) was defined as a signal-to-noise ratio (S/N) of 3:1.

LLOQs were 0.5, 1.5 and 1.0 μg mL^-1^ for INH, PZA, and RIF respectively. LODs were calculated as 1/3 of LLOQs.

*Method Application*

The presented method in this article was successfully employed in a clinical setting to evaluate the plasma levels of isoniazid, pyrazinamide, and rifampin obtained from TB patients for the purpose of therapeutic drug monitoring (15).

*Stability*

The results of all stability tests indicated that the target compounds in plasma samples were stable during the sample storage, preparation, chromatographic analysis, and freeze thaw process. Therefore, the method can be used for the routine analysis of the compounds in plasma.

**Table 1:** Summary of the precision and accuracy of the method

	**Precision (%)**		**Accuracy (%)**
	**Inter-day**	**Intra-day**	
Isoniazid	2.7–11.5	5.2–8.0	96.5–99.1
Pyrazinamide	2.6–4.0	2.1–9.3	92.5–106.6
Rifampin	3.6–8.9	3.5–4.0	91.8–104.8

**Figure 1:**
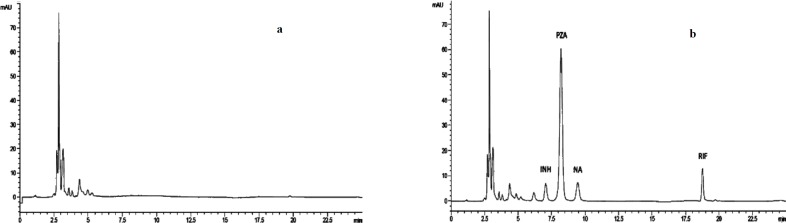
Representative chromatograms of (a) a blank plasma sample, (b) a plasma sample from a patient receiving 300 mg INH, 600 mg RIF, 2000 mg PZA, and 1200 mg EMB spiked with NA. INH: Isoniazid; EMB: Ethambutol; PZA: Pyrazinamide; RIF: Rifmapin; NA: Nicotinamide

## Discussion

Therapeutic drug monitoring in treatment of tuberculosis will help clinicians to achieve successful therapy and minimize toxicity by optimizing the dosage and administration for anti-tuberculosis drugs (15, 16).

Standard protocols for the treatment of tuberculosis consist of, at least, three drugs to avoid rapid development of resistance and treatment failure. 

TDM study for such combination therapy requires a very efficient chromatographic separation method which is capable of accurate determination of each component of the treatment regimen.

The present study was aimed at developing a simple, rapid, and sensitive HPLC method for determination of isoniazid, pyrazinamide, and rifampin in plasma and the application of this method for TDM study in a number of patients infected with *M. tuberculosis*.

Several chromatographic methods have been reported in literature based on HPLC separation of anti-tuberculosis drugs in human plasma. In most of the reports, Sample preparation usually involved a protein precipitation with trichloroacetic acid or organic solvents. However, our study demonstrated that trichloroacetic acid is detrimental for rifampin and the recoveries of rifampin were very low. Different other deproteinization procedures were found to be the addition of acetonitrile along with the zinc sulfate solution.

Reversed (C18) stationary phase was used to achieve the proper separation of the three analytes (INH, PZA, and RIF), and the internal standard (nicotinamide). Since the three target analytes have distinctly different partition coefficients, different chromatographic behaviors were observed for the three compounds of interest.

In order to obtain the shortest run time and the optimum peak symmetry, several chromatographic parameters, including the type of stationary phase, the components and composition of mobile phase, the flow rate, and the elution mode were optimized.

The best stationary phase was found to be C18 column with the particle size of 5 μm. This is the most common stationary phase in HPLC analysis. This method is therefore preferred over the method reported by Zhou *et al.* in which simultaneous determination of INH, PZA, and RIF has been achieved on a C12 stationary phase using heptansulfonic acid as an ion pair agent for optimum separation (13).

A gradient was used starting with 5% methanol for the first 12 min, where after it was increased to 95% methanol within 3 min to 15 min, the water was decreased to 5%. The latter composition was maintained for a further 8 min until the end of the run at 23 min.

The UV detector was adjusted to operate at 254 nm for the first 15 min of the run time for monitoring of INH and PZA and the wavelength was then switched to 336 nm to get the best response for rifampin and minimize the chromatographic interferences.

After providing written informed consent, the method was applied to the real samples from the patients who had received the three drugs, in which no endogenous interferences were observed at the retention time of the three analytes of interest and internal standard (17, 18).

Our method has the advantage of simultaneous determination of INH, PZA, and RIF in a single chromatographic run and to the best of our knowledge, this is the first real simultaneous determination of the three analytes in a single run using an HPLC-UV method. A representative chromatogram is presented in Figure 1.

The only report which claims the simultaneous determination of INH, PZA, and RIF using HPLC-UV method is by Unsalan *et al.* In this report it requires two separate sample preparations procedures, one for PZA together with RIF and the other for INH only. The authors declare that INH may not be observed in the desired concentration for human plasma under the conditions that is optimum for PZA and RIF (11).

Another point which is worth to notice is that no buffer was used in either solvent A or B in our method. In fact, we found that the existence of buffer in mobile phase will substantially increase the column pressure which results in column blockade probably due to the buffer salt precipitation when the organic solvent is increased in the mobile phase. In order to optimize the speed of gradient elution and achieve the best run-to-run repeatability of retention times for INH, PZA, and RIF, we used two column volumes of eluent (1.5 mL min^-1^ for 3 min). There was no need to allow 10-15 column volumes of initial eluent to pass through the column to obtain repeatable results.

This may be somewhat surprising, but very practical and it is in agreement with the elegant report by Schellinger *et al.* who indicate that conditioning a column to provide acceptable run-to-run repeatability in retention time does not necessarily require full equilibration of the column by the initial eluent (18). Recent studies on TDM of patients with MDR-TB also showed the same results (19, 20).

## Conclusion

In this study, a sensitive and specific HPLC-UV method for the simultaneous determination of three first line anti-TB drugs (INH, PZA and RIF) in human plasma was developed. The method was fully validated with respect to specificity, accuracy, and precision.

The proposed method is simple, inexpensive, and stable using a C18 Column coupled to a UV detector, making it a unique and powerful technique for therapeutic drug monitoring in TB patients.
